# An immunostaining-based approach for assessing myocardial viability in the infarcted mouse hearts

**DOI:** 10.3389/fcvm.2025.1598314

**Published:** 2025-06-03

**Authors:** Weili Ouyang, Xueqing Liu, Zheheng Ding, Yanan Ji, Jianfeng Zhao, Hongtao Zhu, Weidong Wu, Zhaoping Ding

**Affiliations:** ^1^ Department of Cardiology, The People’s Hospital of Danyang, Affiliated Danyang Hospital of Nantong University, Danyang, China; ^2^ Institute of Biochemistry and Molecular Biology II, Medical Faculty and University Hospital Düsseldorf, Heinrich-Heine-University of Düsseldorf, Düsseldorf, Germany; ^3^ Department of Anesthesiology, The People’s Hospital of Danyang, Affiliated Danyang Hospital of Nantong University, Danyang, China; ^4^ Institute of Molecular Cardiology, Medical Faculty and University Hospital Düsseldorf, Heinrich-Heine-University of Düsseldorf, Düsseldorf, Germany

**Keywords:** myocardial viability, cardioprotection, cardiomyocytes, acute myocardial infarction, immunostaining, cardiac troponin I, TTC staining

## Abstract

**Introduction:**

With the growing need for reliable and precise detection of cell viability in spatial biology, we introduce an antibody-based staining of cardiac troponin I (cTnI) as a simple yet valuable tool for delineating cardiomyocyte viability in the early stages of myocardial infarction (MI).

**Methods & Results:**

In circulation, cTnI was found to be the most abundantly released biomarker within the first 24 h after MI. In heart sections, partial depletion of cTnI staining was observed within dying cardiomyocytes as early as 6 h, with almost absence by 24 h despite of preserved membrane integrity. In contrast, staining for other sarcomeric proteins, such as troponin T and *α*-actinin, remained detectable for several days until immune cells infiltration occurred. We further validated the rapid loss of cTnI staining by cross-verifying *in-vivo* and *ex-vivo* measurements. Notably, cTnI-stained sections showed precise overlap with TTC-stained images at the cellular level and showed a highly consistent pattern of cardiomyocyte distribution and infarct area (*r*² = 0.96) when compared to *in-vivo* measurements using manganese-enhanced magnetic resonance imaging (MEMRI).

**Conclusion:**

These findings highlight the coordinated, stepwise breakdown of sarcomeric proteins following ischemic injury in the mouse heart and underscore the utility of antibody-based cTnI staining as a valuable tool for early myocardial viability assessment and infarct area detection with high spatial resolution.

## Introduction

Cardioprotection remains one of the most compelling and widely pursued strategies to mitigate ischemia/reperfusion injury after myocardial infarction (MI) (1). Recently, stem cell-based therapy has been shown to confer cardioprotective effects that preserve ischemic cardiomyocytes from apoptotic injury (2, 3). Transplantation of multipotent stem cells into the damaged heart, unlike cardiac-committed cells, which integrate persistently into the host myocardium (4), primarily exert protective effects by secreting soluble factors beneficial to the ischemic myocardium before they disappear, likely through a potent cellular postconditioning mechanism (5, 6).

In all studies focused on cardioprotection, the primary endpoint must be a quantitative assessment of cardiomyocyte viability, a key scientific measure for evaluating protective efficacy (7). In this context, a reliable and reproducible method for assessing myocardial viability is essential—not only for evaluating the effectiveness of treatment strategies but also for elucidating the mechanisms underlying the ischemic tolerance of surviving cardiomyocytes in the infarcted heart.

The majority of experimental studies have relied on triphenyl-tetrazolium chloride (TTC) staining to identify viable tissue, providing an accurate measure of cell viability both in tissue sections (8) and at the cellular level (9). However, despite its advantages, TTC method is typically used in gross histology and, even when applied in microscopic imaging, cannot be easily integrated with other assays for mechanistic studies. With the growing need for viability detection in modern spatial analysis using antibody-based multiplexing and multi-omics approaches (10), a reliable and standardized antibody-based method for assessing myocardial viability is urgently needed, particularly in the early stage of MI when salvaging cardiomyocytes is critical and highly desirable.

In the infarcted heart, the coordinated, stepwise breakdown of sarcomeric proteins is tightly regulated by distinct intrinsic mechanisms, resulting in the sequential depletion of contractile proteins in dying cells. Cardiac troponins, the most susceptible targets of proteolysis, are rapidly degraded by calpains, caspases, and the ubiquitin-proteasome system (UPS), which are activated by calcium leakage resulting from membrane disruption (11). In contrast, other cardiac proteins, such as myosin heavy chain, actin, and titin, undergo progressive degradation primarily during the subacute phase, coinciding with immune cell infiltration. Accordingly, the early loss of troponins may serve as a key histological marker for delineating cell viability in the infarcted heart (12).

The present study aims to address two fundamental questions regarding the feasibility of antibody-based staining for viability detection. First, which troponin is the most vulnerable and the earliest to disappear in necrotic/apoptotic cells? Second, does the immunostaining method accurately correlate with TTC staining and *in-vivo* measurements? To answer these questions, we tested systemically several antibodies of cardiac proteins to evaluate their potentials and identified cardiac troponin I (cTnI) as a valuable marker for the early detection of cardiomyocyte viability in the infarcted murine heart.

## Materials and methods

All animal experiments were approved by the Institutional Animal Care and Use Committee of Nantong University, China, and conducted in accordance with the ARRIVE guidelines and the NIH *Guide for the Care and Use of Laboratory Animals*. Male adult C57BL/6J mice (20–25 g, purchased from Jackson Laboratory) were used in this study. All animals were provided with a standard chow diet and had *ad libitum* access to tap water.

### Cardiac ischemic injury

Experimental myocardial infarction (MI) was induced by transiently tying off the left anterior descending coronary artery (LAD) as previously described (13). Briefly, mice were anesthetized by inhaling 1.5% v/v isoflurane (Shandong Keyuan, China) via a tracheal cannula (20G, Vasofix, Braun), and placed on a pre-warmed surgical table maintained at 37°C. Two electrodes were attached to an electrocardiograph (ADInstrument) for continuous ECG monitoring. A thoracotomy was performed at the fourth intercostal space, lateral to the sternum. After identifying the LAD on the heart surface, a polypropylene suture (8-0 Prolene®, Ethicon) was passed underneath the vessel, marginally below the tip of the left auricle. The LAD was protected by a small piece of soft tissue pad (1 × 1 × 2 mm) on the vessel, while the suture was securely tightened to obstruct coronary blood flow, inducing a transient myocardial ischemia (MI) downstream the ligation. Successful occlusion was confirmed by surface blanching and ST segment elevation in ECG registration. LAD occlusion was maintained for 50 min before coronary perfusion was restored by loosening the ligature. The chest was then closed in two layers: one through the muscle and another through the skin. The animals were weaned from mechanical ventilation and placed in a warm, oxygen-enriched environment until fully recovery of physical activity. Each animal received an intraperitoneal injection of butorphanol (1 mg/kg, Hengrui, China) for analgesia.

### Heart sample preparation and cryosectioning

Twenty-four hours after MI surgery, the animal was euthanized by cervical dislocation under deep anesthesia (3% v/v isoflurane). The heart, along with the lungs and surrounding tissue, was immediately excised from the thoracic cavity and transferred to a Petri dish filled with ice-cold phosphate-buffered saline (PBS). Under a stereomicroscope, the heart was carefully trimmed to remove non-cardiac tissues. A blunt, curved cannula (23G) was inserted through the opening of the right and left atria into the ventricular cavities, allowing for injection of Tissue-Tek® O.C.T. Compound (Sakura, Umkirch, Germany) to preserve the heart's natural four-chamber structure. The heart was then positioned in as cryomold filled with Tissue-Tek® in a vertical position, aided by a cooling bath filled with 2-methylbutane with a temperature of −30 to −40°C to minimize ice crystal formation. Finally, the sample block was stored at −80°C, ready for the next step of cryosectioning.

### Immunostaining in heart sections

The tissue block was mounted onto a cryostat chuck using Tissue-Tek® and the heart was cut at a thickness of 7 µm at a temperature at approximately −23°C to −25°C. Slides were then air-dried to enhance tissue adherence and subsequentially fixed with Zamboni's fixative (2% paraformaldehyde in PBS with picric acid, Morphisto, Germany) for 10–15 min at room temperature.

Immediately after fixation and three washes with PBS, both Hematoxylin and Eosin (H&E) staining and immunohistological analysis were performed. For immunostaining, the tissue slides were first incubated with 5% normal goat serum (NGS) for 60 min before the addition of primary antibodies. The following antibodies were used in this study: mouse anti-α-actinin (ACTN2, Cat. No.: A7811, Thermo Fisher), mouse anti-cardiac troponin T (cTnT, Cat. No.: MA5-12960, Thermo Fisher), rabbit anti-cardiac troponin I (cTnI, Cat. No.: 21652-1-AP, Proteintec), rat anti-laminin (Cat. No.: ab11576, abcam), goat anti-myoglobin (Mb, Cat. No.: sc-31110, Sant Cruz) and rat anti-CD68 (FA-11, Cat. No.: ab53444, Abcam). All antibodies were diluted 1:200 in incubation buffer (PBS with 1% NGS and 0.1% saponin), and an equal volume of buffer without antibody was used as a control. Slides were incubated overnight at 4°C. The next day, the sections were washed three times with PBS and incubated with secondary antibodies (Cy3- or FITC-conjugated goat anti-rabbit or rat, 1:400, Cat. No.: 111-547-003 and 112-165-003, Jackson ImmunoRes and Cy3-conjugated goat anti-mouse Fab IgG, 1:400, Cat. No.: 115-007-003, Jackson ImmunoRes) at room temperature for 1 h in darkness. For staining of the primary antibodies raised in mice (cTnT and ACTN2), endogenous IgG cross-reactivity was blocked using anti-mouse Fab fragment saturation prior to the addition of the primary antibodies. Cell nuclei were counterstained with 4′,6-diamidino-2-phenylindole (DAPI), and slides were mounted with Prolong™ Gold (Cat. No.: 1626789, Mol. Probes). Images were acquired using a fluorescence microscope (Olympus MX61, Japan) and analyzed with ImageJ software (version 1.53a, NIH, USA).

### Microscopic TTC assay

Photomicrographs were obtained from the TTC-stained heart using a refined TTC assay, as previously reported (6, 9). Briefly, the heart was harvested 24 h after MI surgery and retro-perfused three times consecutively with 1 ml of TTC solution (1% in PBS) via an aortic cannula, each for 5 min, followed by overnight incubation in TTC solution at 4°C. The next day, the heart tissue embedded in Tissue-Tek® and sectioned transversely at a thickness of 10 µm. The tissue slides were subject to immune co-staining as described above. Images were captured using a digital camera (UC30, Olympus, Japan) operated with CellSens® software (Olympus, Japan).

### Detection of cardiac markers by ELISA kits

To monitor the dynamics of serum cardiac markers (cTnT, cTnI and ACTN2), blood samples were collected from the intraorbital vein at 3 and 6 h after MI surgery under transient anesthesia (2% isoflurane). Additional samples were obtained from the inferior vena cava at 24 and 48 h, just before the mice were euthanized for heart sample preparation. Serum samples (minimum volume: 50 µl) were prepared, and troponin concentrations were measured using either an electrochemiluminescence immunoassay (ECLIA; e411, Roche Elecsys) for cTnT or a chemiluminescent microparticle immunoassay (CMIA; i1000SR, Abbott Architect) for cTnI, both performed in the hospital's routine clinical laboratory. Serum ACTN2 levels were assessed using an ELISA kit (NBP2-66418, Biotechne, USA) following the manufacturer's instructions.

### Detection of cardiac apoptosis by TUNEL assay

Cardiac apoptosis was assessed using a commercial TUNEL detection kit (TUNEL Andy Fluor 594, ABP Bioscience) following the manufacturer's instructions (6). Briefly, cryosections of heart samples were fixed and permeabilized with 0.2% Triton-PBS for 30 min at room temperature. The samples were then incubated with labeled dUTP and TdT enzyme for 2 h at 37°C. After three washes with PBS, the slides were incubated with Andy Fluor™ 594-Streptavidin for 60 min at room temperature. All images were digitized and analyzed as described above.

### *In-vivo* measurement of myocardial viability

To compare myocardial viability *in vivo*, we implemented a noninvasive manganese-enhanced magnetic resonance imaging (MEMRI) assay in the same heart used for immunostaining. The MEMRI measurements were performed shortly before the heart sample was harvested for immunostaining. In brief, a MnCl_2_ mixture (60 µl, 0.01%) with calcium gluconate (25 µl, 0.03%) was prepared and sonicated until full dissolved. MEMRI was then conducted immediately after intravenous injection of the solution, using a 400 MHz Bruker AVANCE III 9.4T wide-bore nuclear magnetic resonance spectrometer (Bruker, Germany) operated by ParaVision 5.1 software. Cardiac images were acquired with the Bruker Microimaging unit (Mini 0.5), equipped with a 30 mm birdcage resonator. Six to eight contiguous ventricular short axis slices (thickness 1 mm) were acquired and stored for each heart. Off-line analysis was performed with the region of interest (ROI) tool in Bruker ParaVision to manually delineate the negative area in MEMRI measurement in end-systole phase of each slice.

### Statistical analysis

Data were presented as mean ± standard deviation. Two-way analysis of variance (ANOVA) followed by Bonferroni was used to compare the serum concentrations of cardiac markers at different timepoints. Differences were considered significant at *p* < 0.05. The Prism software package (version 9.0) was used for the statistical analysis.

## Results

### Cardiac troponin I as a reliable marker defining the viable cardiomyocytes

**Figure 1:**
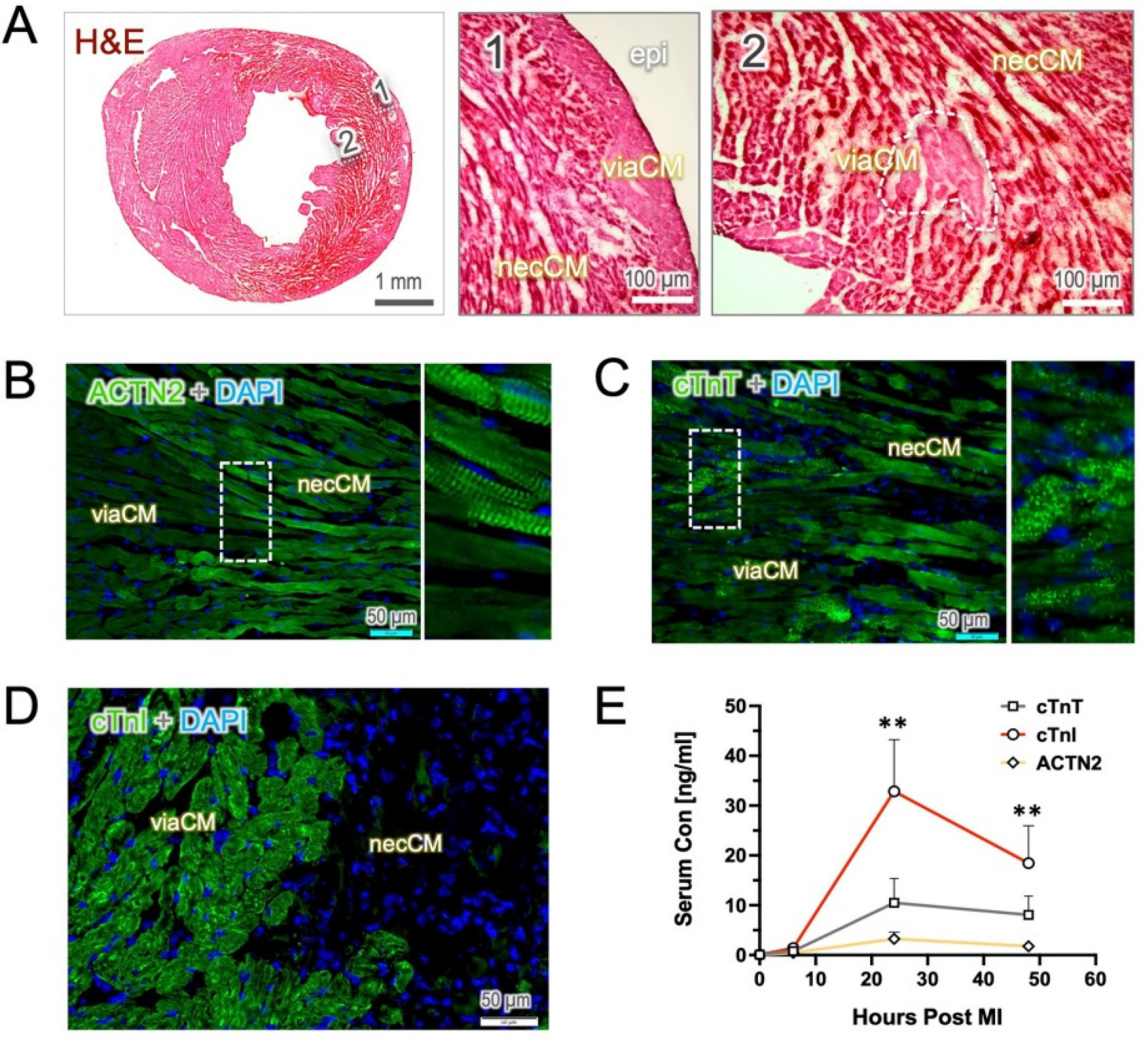
Histological staining of the heart sections and injury markers in the blood. Hematoxylin & eosin (H&E) staining and immunostaining were performed on the tissue sections from the mouse heart harvested 24 h post-MI. **(A)** The H&E image reveals a hypereosinophilic appearance in the necrotic tissue (necCM) and a loss of tissue integrity. In contrast, viable cardiomyocytes (viaCM) exhibit moderate staining and are primarily located in the epicardial area (epi, area 1) as well as within the infarct zone in small clusters (area 2). **(B,C)** In images of anti-ACTN2 and cTnT staining, viaCM show weak staining, whereas necCM exhibit enhanced fluorescence intensity. Notably, ACTN2 staining highlights sarcomeric striations in the dying cells, and cTnT-positive staining appears in multiple vesicles. **(D)** cTnI-positive staining is predominantly found in viable cardiomyocytes, whereas necrotic cardiomyocytes show little to no staining. **(E)** The dynamics of the three injury biomarkers in blood follow a similar pattern, with levels peaking mostly at 24 h post-MI. Interestingly, serum cTnI remains significantly elevated at 24- and 48 h post-MI compared to cTnT and ACTN2. ** indicates *p* < 0.01.

Cardiac ischemia leads to a severe disruption of tissue integrity in the ventricular wall due to the lack of blood supply, leaving only the endocardial and sub-epicardial portions viable. In H&E staining, nonviable or necrotic cardiomyocytes (necCM) can be morphologically distinguished from viable cardiomyocytes (viaCM), as dying cells often exhibit cell body shrinkage, loss of cell structure and an increased affinity for eosin staining (hypereosinophilia, Figure 1A). However, clear identification of the viaCM remains challenging, even at high magnification of the H&E images.

To specifically define viable cardiomyocytes in infarcted hearts, we used antibody-based strategy to stain tissue with cardiac markers in the acute phase (24 h post-MI, 24 h), as the loss of sarcomeric proteins (e.g., ACTN2, troponin T, and I) may serve as an indirect indicator of myocardial necrosis. In the heart 24 h post-MI, only minimal autofluorescence was observed in the antibody controls in both the FITC and Cy3 channels, primarily within the infarcted myocardium (Supplementary Figure S1). Notably, immunostaining with ACTN2 revealed that, compared to viable cardiomyocytes (viaCM), necrotic cardiomyocytes (necCM) exhibited surprisingly hyper-reactivity to ACTN2 but still retained typically the striation pattern as mature cardiomyocytes. In contrast, the viaCM displayed relatively lower fluorescence intensity than the necCM, while the tissue integrity was maintained (Figure 1B). A similar observation was confirmed in anti-cTnT staining, which demonstrated an enhanced affinity for the cTnT antibody and the presence of numerous cTnT-containing vesicles in the necCM (Figure 1C). The viability of weak-staining cardiomyocytes was further confirmed by microscopic TTC staining as a gold-standard, showing an exact overlay between those cardiomyocytes and the TTC-positive cells (Supplementary Figure S2). Notably, at this stage, TTC-negative cardiomyocytes (suggestive of necCM) still exhibited an intact cell membrane, as confirmed by laminin immunostaining (Supplementary Figure S2).

In contrast, staining with cTnI clearly delineated the viaCM, creating a stark contrast that effectively distinguished viable from nonviable cardiomyocyte which stained completely negative in the sections (Figure 1D). The absence of cTnI staining was likely due to the rapid elimination of cTnI protein in damaged cardiomyocytes. In line with histology, serum cTnI concentrations were found to be significantly higher than those of cTnT and ACTN2, although all markers peaked 24 h after the onset of MI (Figure 1E).

**Figure 2:**
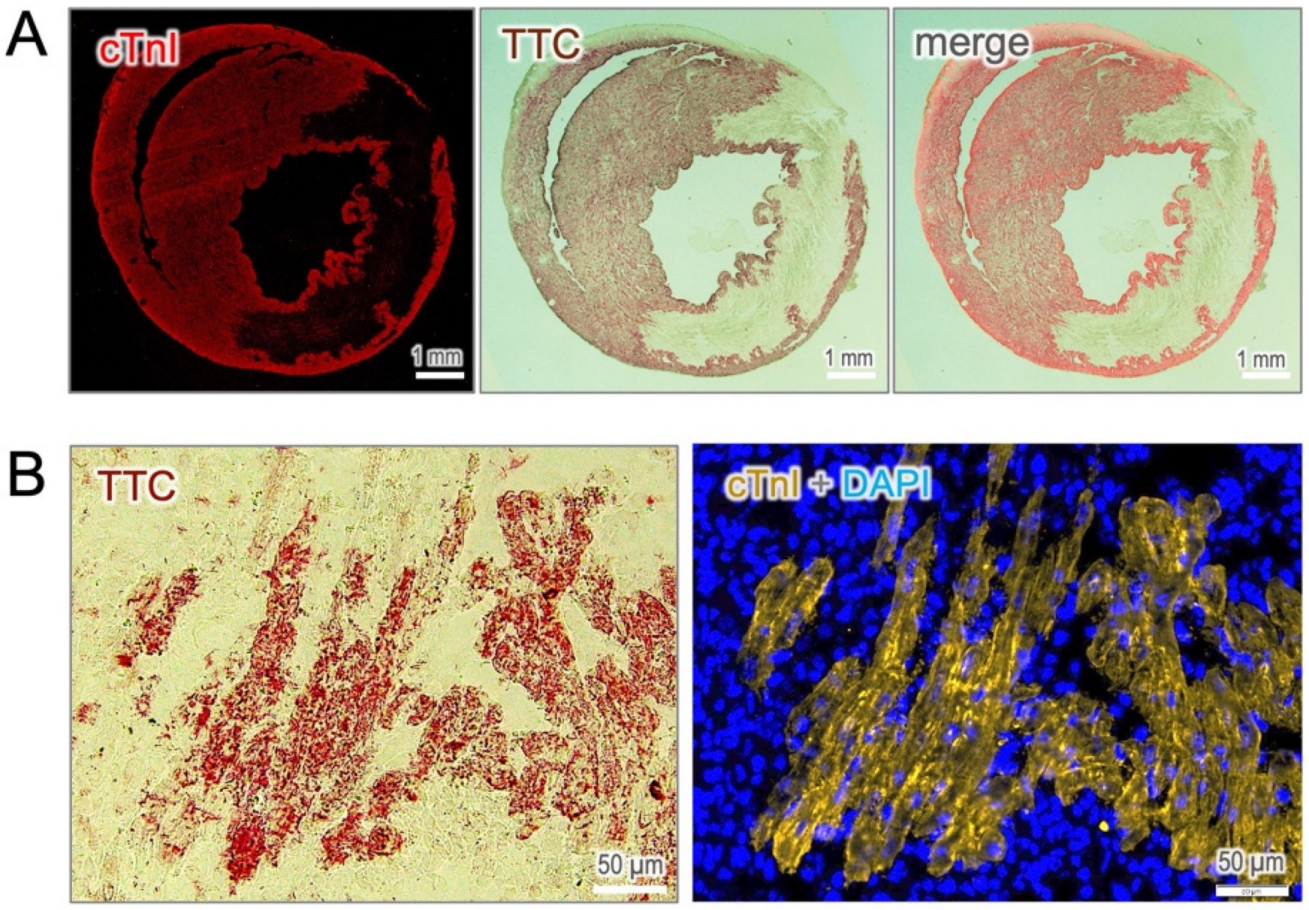
Validation of cTnI-positive cells with TTC staining. **(A)** Co-staining of cTnI with TTC reveals a precise overly between viable cardiomyocytes identified by both approaches. **(B)** At the cellular resolution, cTnI-positive cells correspond exactly with TTC-positive staining, which serves as the gold standard for assessing cell viability.

To confirm that cTnI-staining truly identifies viable cardiomyocytes, we performed co-immunostaining of cTnI with the microscopic TTC assay, a gold standard method for assessing cellular viability. As shown in Figure 2A, the cTnI-positive area precisely matched the TTC staining, while the infarct zone was homogenously negative regardless of infarct regions. At the cellular level, the viable cardiomyocytes, which preserve potent dehydrogenase activity, convert colorless TTC into red precipitates that corresponded precisely with positive cTnI-staining (Figure 2B). These findings establish cTnI-based immunostaining as a reliable and valuable histological approach for the identification of viable cardiomyocytes in the infarcted heart.

### Early breakdown of troponin I in the injured cardiomyocytes

**Figure 3:**
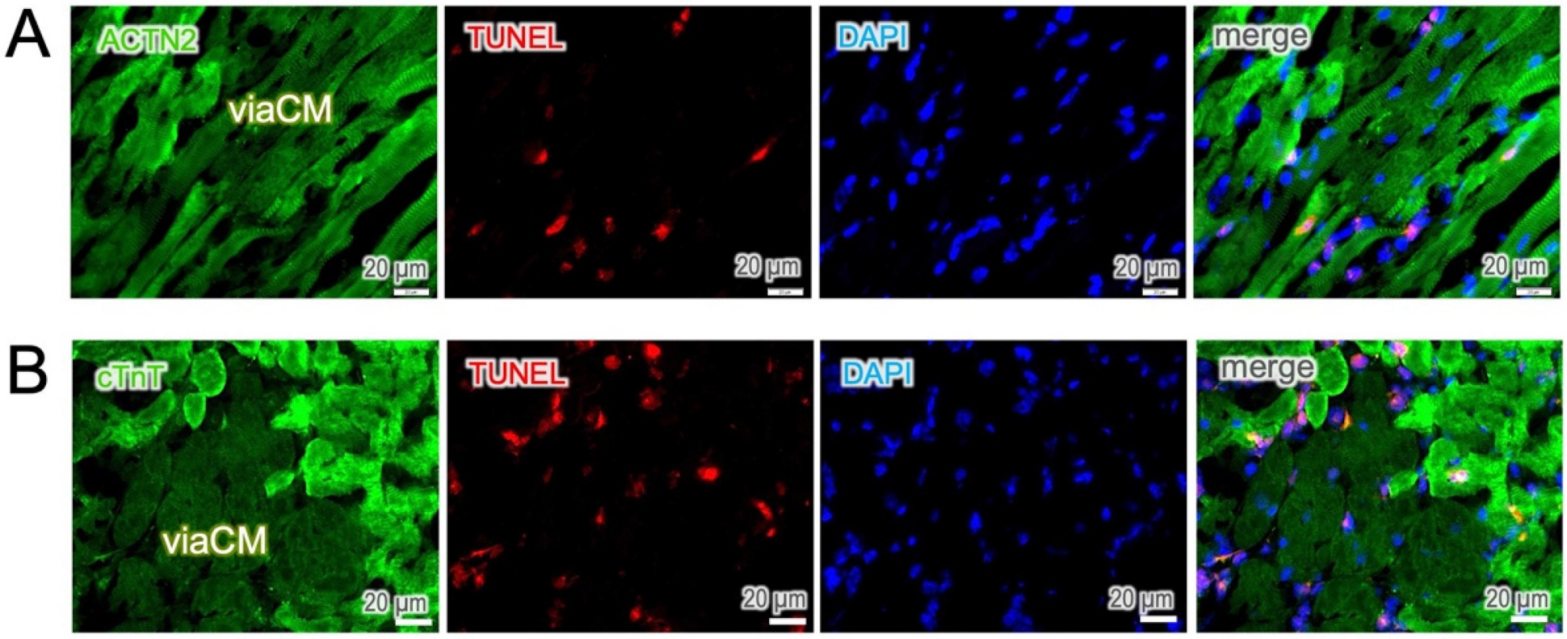
Apoptosis occurs predominately in hyperreactive cells. **(A,B)** TUNEL staining was used to identify apoptotic cells. TUNEL-positive cells were found predominantly in interstitial cells and in ACTN2- and cTnT-hyperreactive cardiomyocytes, whereas moderately stained cells, known as viable cardiomyocytes (viaCM), were largely negative.

The hyperactive staining of ACTN2 and cTnT is likely due to the nonspecific binding of antibodies to cell debris in dying cells. Therefore, we performed a TUNEL assay on the sections to detect DNA fragments in cells undergoing apoptosis. Positive TUNEL staining was predominantly observed in the cells of high fluorescence intensity and, in parallel, loss of cell integrity. In contrast, viaCM remained TUNEL-negative in both ACTN2 (Figure 3A) and cTnT (Figure 3B) staining, signifying an antibody hyper-reactivity in dying cardiomyocytes.

**Figure 4:**
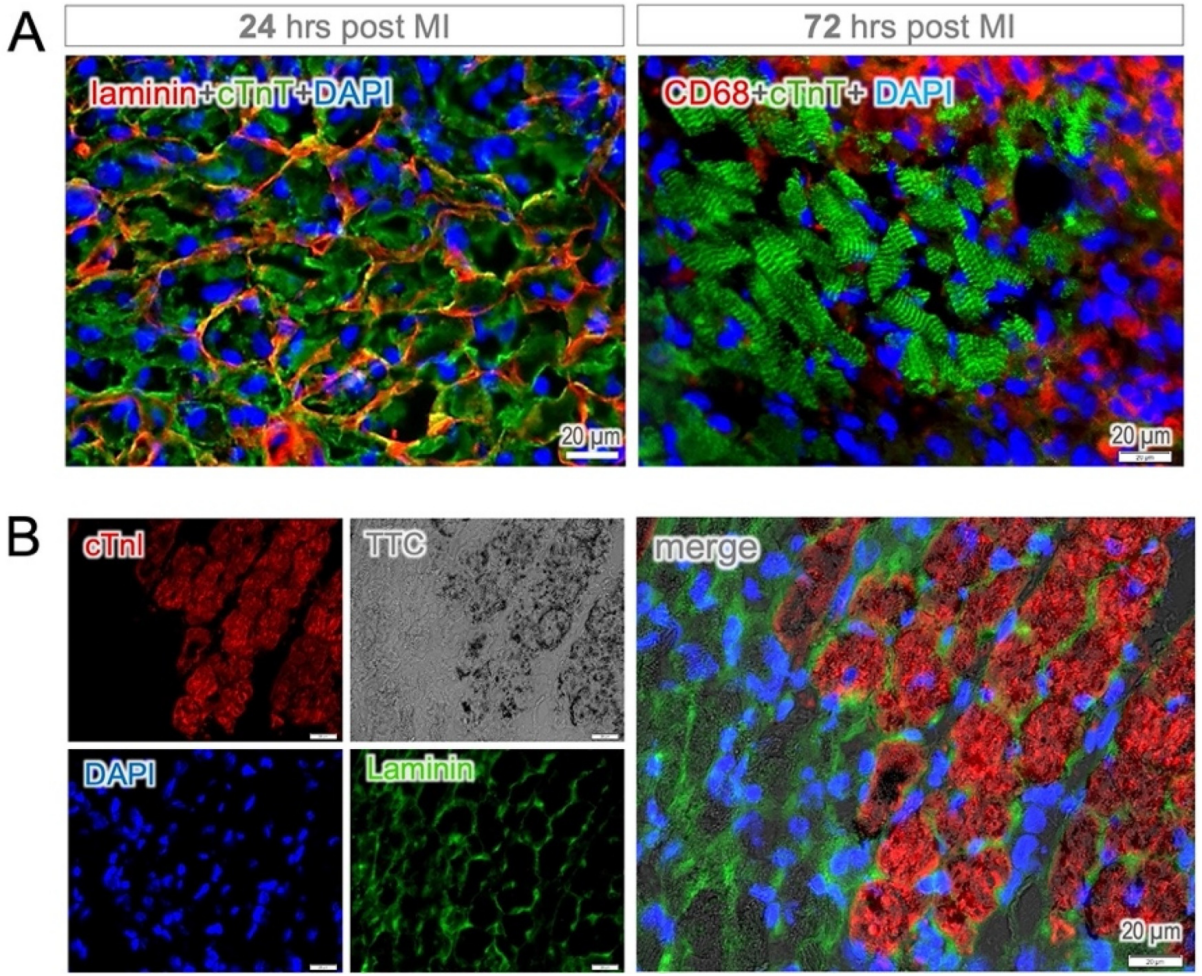
Protein degradation and membrane integrity. **(A)** Cytoplasmic cTnT-positive staining was observed in dying cardiomyocytes while the cell membrane (defined by laminin staining) remained intact 24 h post MI (left panel). The cTnT-positive staining of cell debris was found to persist up to 72 h, primarily in the infarct core, which was surrounded by intense infiltration of phagocytic cells (CD68-positive, right panel). Note both the membrane and cytosolic locations of CD68-staining in macrophages. **(B)** Despite membrane integrity, the disappearance of immune positivity was observed in heart tissue sections 24 h post MI. Notably, cTnI-positive staining corresponded at the cellular level to TTC staining, as indicator of cell viability.

It became more evident when the cell membrane was visualized by using laminin staining, revealing the disorganization of the sarcomeric structure in dying cardiomyocytes (left panel in Figure 4A). However, cTnT-positive staining of cell debris remained detectable for up to 72 h, primarily in the infarct core, until clearance mediated by phagocytic cells (CD68-positive) occurred (right panel in Figure 4A). In contrast, cTnI elimination was found to occur at a much early stage while the cell membrane was still intact, resulting in a complete loss of staining in nonviable cardiomyocytes (TTC-negative, Figure 4B). These findings suggest that time-dependent mechanisms may be involved in the breakdown of sarcomeric proteins in dying cardiomyocytes, with cTnI being the earliest structural protein to undergo degradation in dead cells. This unique feature of rapid elimination allows for early histological discrimination of cell viability using cTnI-staining, even as early as six hours post-MI (Supplementary Figure S3).

### Comparison of cTnI-staining approach to *in-vivo* MEMRI

**Figure 5:**
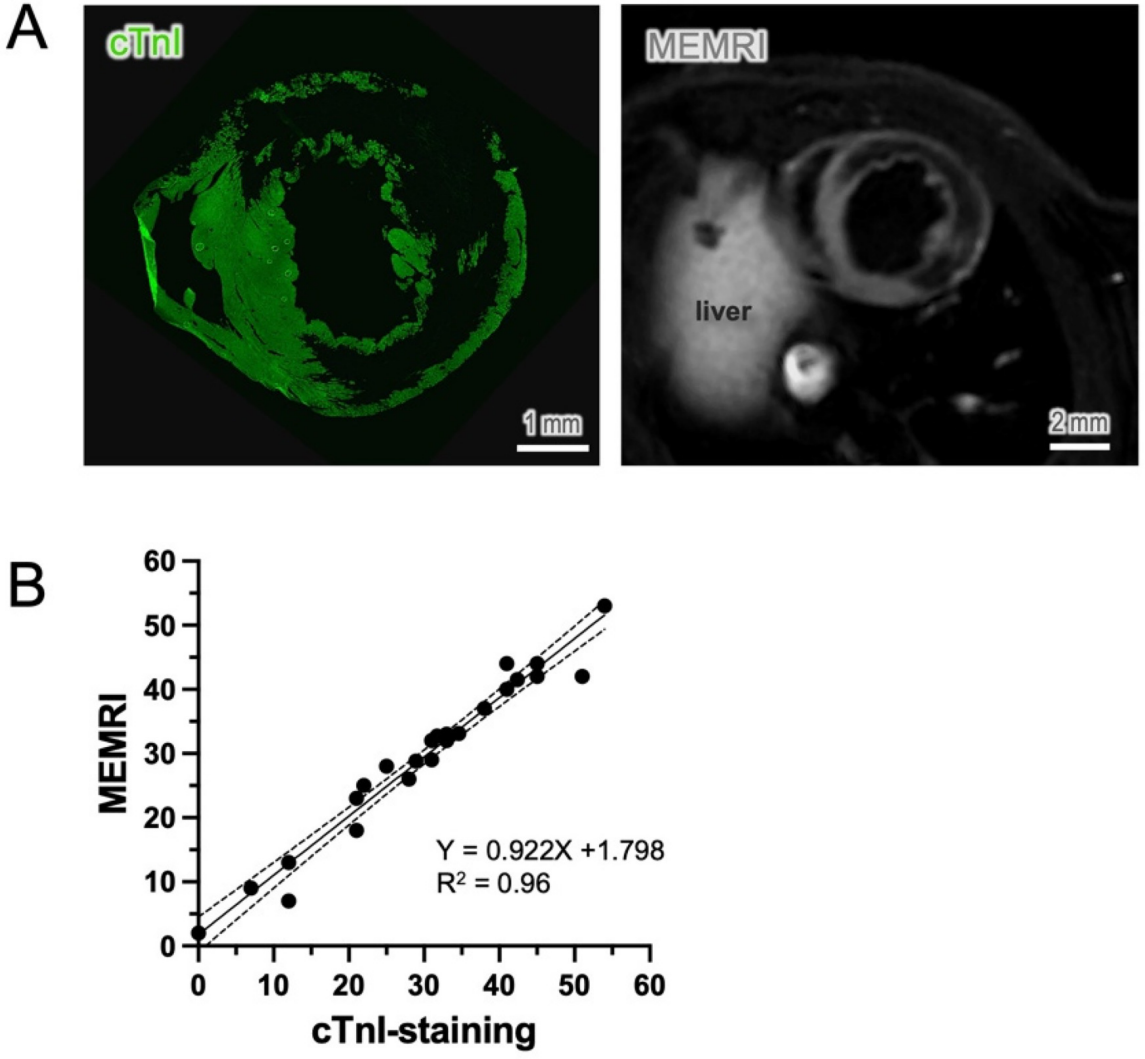
Comparison of cTnI staining to *in-vivo* measurements. **(A)** cTnI immunostaining showed an almost identical distribution pattern of viable cardiomyocytes to the cardiac images derived from *in-vivo* manganese-enhanced magnetic resonance imaging (MEMRI). **(B)** The nonviable area, known as the infarct area, was compared at a similar level and showed a significant correlation between the two methods (*n* = 6, sections = 24, *p* < 0.01).

To verify the suitability of choosing cTnI as a viability maker, we compared the cTnI-staining method to the well-known *in-vivo* measurements by MEMRI in the same animal. At comparable section levels, both cTnI staining and the *in-vivo* imaging (Figure 5A) exhibited a similar pattern and distribution of viable myocardium, along with a corresponding infarct area devoid of vital cardiomyocytes. Both methods exhibited highly correlated detection of infarct area at the comparable sections (*r*^2^ = 0.96, 24 slides from 6 hearts, Figure 5B). By contrast, staining with cTnT and ACTN2 exhibited strong residual fluorescence signals in the infarct region and a low viable-to-necrotic signal ratio, which makes it difficult to delineate the infarct area (Supplementary Figure S4). This direct visual comparison supports the rationale for selecting cTnI as a more suitable marker for early detection of cardiomyocyte viability and infarct size.

## Discussions

Modern spatial biology provides a powerful approach for high-throughput analysis of tissue transcriptomics and proteomics at single-cell resolution. In this context, a simple and integrable method for assessing cell viability is highly desirable. In the present study, we establish an antibody-based immunostaining technique to assess cardiomyocyte viability in the infarcted mouse heart. Our results demonstrate that cTnI, which is rapidly degraded in dying cardiomyocytes, serves as an early histological marker for distinguishing between dying and viable cells. This technique enables the visualization of cellular viability and may serve as a valuable tool for uncovering survival mechanisms following ischemic injury.

The use of antibody-based immunostaining to distinguish viable from non-viable cardiomyocytes was introduced many years ago (14) and has become particularly important in forensic medicine, where it is often used to detect postmortem myocardial injury (15). In this context, selecting appropriate cardiac protein targets at different time points is crucially important. In damaged cardiomyocytes, sarcomeric proteins undergo a sequential degradation process, beginning with the most vulnerable components and progressing toward complete structural disintegration. The earliest protein to be degraded is cTnI, which is highly susceptible to proteolysis (11). Shortly after myocardial injury, calcium influx resulting from membrane disruption activates calpains, caspases, and the ubiquitin-proteasome system (UPS), leading to the destabilization of the troponin–tropomyosin complex and the rapid breakdown of cTnI (12). At later time point, caspases further contribute to cTnI degradation, generating smaller fragments that enter circulation and serve as key biomarkers of cardiac injury (16).

In the present experiments, we found that serum cTnI was already detectable in the blood as early as 6 h and peaked at 24 h after MI. In cardiac tissue, an inverse relation with the serum cTnI is expected. At 6 h post-MI, a substantial reduction in immunopositivity was observed in necCM (Supplmentary Figure S2), readily allowing for the distinction between viaCM and necCM. Nevertheless, due to the partial remnants of cTnI protein in non-viable cells at this stage, a clear determination of cell viability may require additional references, such as cell integrity, unless the signal-to-noise ratio of fluorescence intensity of the staining is sufficiently distinguishable. At 24 h post-MI, when progressive cleavage of cTnI in dying cardiomyocytes is nearly complete, early viability detection becomes straightforward and reliable (Figure 1D). The accuracy of this method was further crosse-validated using two independent approaches. *In vivo*, cardiomyocyte viability was compared to MEMRI method (17), which revealed a nearly identical distribution pattern of viable cardiomyocytes and infarct area in comparable sections of the same animal (Figure 5). *In vitro,* we employed a refined microscopic TTC assay as the gold standard for identifying cell viability at the cellular level. The immunostaining method showed a precise overlap between cTnI-positive cells and TTC staining (Figure 2), underscoring the reliability and accuracy of cTnI immunostaining as a useful tool for detecting cardiomyocyte viability.

Sarcomeric proteins such as ACTN2 and cTnT are targeted only in the subacute phase by calpains and lysosomal enzymes like cathepsins, leading to the complete disassembly of the sarcomere several days after MI. As necrotic debris accumulates, immune cells—particularly neutrophils—infiltrate the damaged myocardium, releasing reactive oxygen species (ROS) and proteolytic enzymes that accelerate protein degradation. Monocyte-derived macrophages follow, engulfing cellular debris and apoptotic neutrophils through phagocytosis (Figure 4A). Professional phagocytes, including macrophages and dendritic cells, clear troponin fragments and sarcomeric remnants while secreting anti-inflammatory cytokines to resolve inflammation and promote tissue remodeling.

In the bloodstream, although ACTN2 and cTnT exhibited similar releasing patterns, we found that these proteins persisted for up to 72 h after MI. In the tissue section, cTnT remained detectable until extracellular matrix proteins such as collagen and laminin were degraded by matrix metalloproteinases (Figure 4 and Supplementary Figure S2). The sustained positive signals with enhanced fluorescence intensity in necrotic myocytes make anti-ACTN2 and -cTnT staining most challenging to determine viability at the early stages (Supplementary Figure S4). This finding raises questions about a recently reported method for detecting early myocardial infarction by targeting F-actin, which likely persists for a relatively long period in the infarcted area (18).

Several other approaches have been introduced in previous publications. Tissue edema and hypereosinophilia were notable in H&E imaging, however, these do not met the prerequisite, as the distinction between the necrotic and the non-necrotic areas remains unclear and judgment tends to be subjective (11). Myoglobin (Mb), an oxygen-carrying protein found exclusively in skeletal and cardiac muscle cells, is thought to be released through the deteriorating cell membrane in necrotic myocardium. Depletion of Mb from the myocardium may therefore serve as a marker for irreversible ischemic injury (19, 20). However, Mb is a diffusible protein that often generates weak background staining in non-myocytes (21), which limits the suitability of Mb as a standard target for assessing cardiomyocyte viability.

Given the high specificity of cTnI-based staining for cardiomyocyte injury and necrosis, this method, beyond its role as a diagnostic readout following MI, can be technically integrated into spatial profiling platforms such as the GeoMX Digital Spatial Profiler (DSP) and CODEX (CO-Detection by Indexing) (22). In the antibody-based multiplexing, cTnI can serve not only as a cell-type marker but also as a state-specific indicator of cardiomyocyte viability. This enables high-resolution insights into single-cell-level relationships within the cardiac tissue microenvironment and may uncover spatially associated signaling events, immune cell infiltration, or survival pathways that impose cardiomyocytes the ability of becoming ischemic resistance, which may ultimately guide the identification of novel therapeutic targets for cardiac protection (23).

## Conclusion

In summary, we here introduce an antibody-based staining technique to assess cell viability in tissue sections. Our results demonstrate that the loss of cTnI is a consistent and prominent feature in dying cardiomyocytes. Therefore, immunohistochemical staining using an antibody against cTnI can be used to visualize cardiac troponin I and document its early loss in histologic sections of the injured myocardium. This method offers a novel approach to not only determine cell identity but also assess cell viability, making it highly valuable for antibody-based spatial multiplexing analysis in heart tissue sections.

## Data Availability

The raw data supporting the conclusions of this article will be made available by the authors, without undue reservation.
