# Supplementary material for: An immunostaining-based approach for assessing myocardial viability in the infarcted mouse hearts
Source: Front Cardiovasc Med. 2025 Jun 3;12:1598314. doi: 10.3389/fcvm.2025.1598314 (PMC12170573; doi:10.3389/fcvm.2025.1598314)
Supplement: Supplementary file 1 [file Datasheet1.pdf]

An immunostaining-based approach for assessing myocardial viability in the infarcted mouse hearts

running title: assessing myocardial viability by cTnI-staining

Weili Ouyang[1*], Xueqing Liu[1*], Zheheng Ding[2], Yanan Ji[1], Jianfeng Zhao[1], Hongtao Zhu[1], Pascal Bouvain[3], Ulrich Flögel[3], Weidong Wu[3#] and Zhaoping Ding[4#]

[1] Department of Cardiology, The People's Hospital of Danyang, Affiliated Danyang Hospital of Nantong University, West Xinmin Rd. 2, Danyang 212300, China.

[2] Institute of Biochemistry and Molecular Biology II, Medical Faculty and University Hospital Düsseldorf, Heinrich-Heine-University of Düsseldorf, Universitätsstr. 1, 40225 Düsseldorf, Germany

[3] Department of Anesthesiology, The People's Hospital of Danyang, Affiliated Danyang Hospital of Nantong University, West Xinmin Rd. 2, Danyang 212300, China.

[4] Institute of Molecular Cardiology, Medical Faculty and University Hospital Düsseldorf, Heinrich-Heine-University of Düsseldorf, Universitätsstr. 1, 40225 Düsseldorf, Germany

Supplementary Figure 1

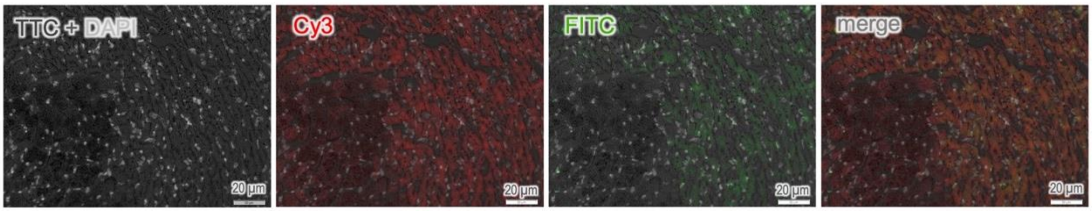

Antibody control staining in the infarcted heart. To validate the specificity and signal accuracy of antibody-based staining in hearts from mice 24 hours post-MI, tissue sections were processed using the same protocol as standard immunostaining, but without primary antibodies. In both the Cy3 and FITC channels, only weak fluorescence signals were observed, primarily in the non-viable myocardium, likely due to an autofluorescence created by the necrotic tissue (TTC-negative). These findings support the feasibility of applying immunostaining in the early infarcted heart, with an acceptable signal-to-noise ratio.

Supplementary Figure 2

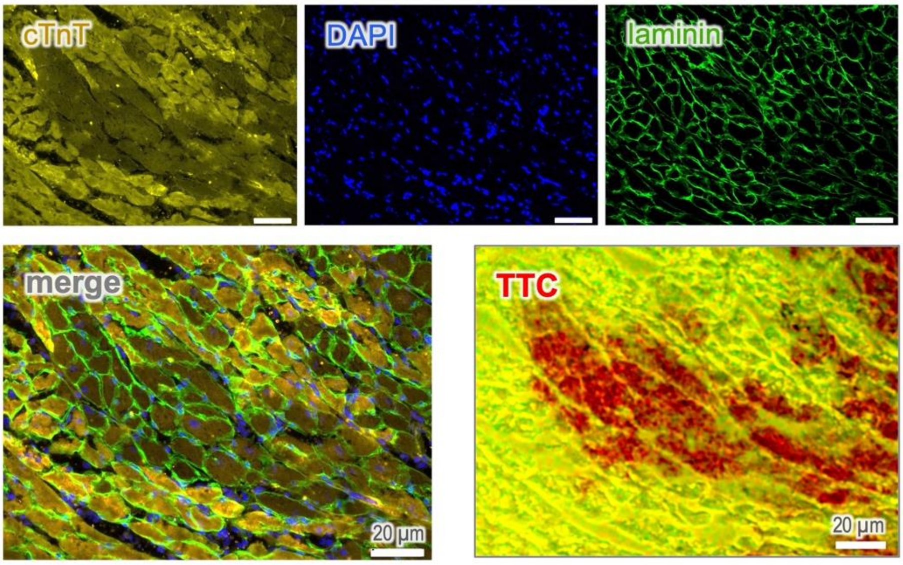

Hyper-reactivity of cTnT immunostaining in necrotic cardiomyocytes. A heart sample was obtained from mice 24 hours post-MI and subjected to TTC and cTnT co-staining. Viable cardiomyocytes, identified by TTC positivity, exhibited moderate staining, whereas necrotic cells showed markedly enhanced fluorescence intensity of cTnT immunostaining. Notably, at this time point, the cell membrane remained entirely intact, as confirmed by laminin staining.

Supplementary Figure 3

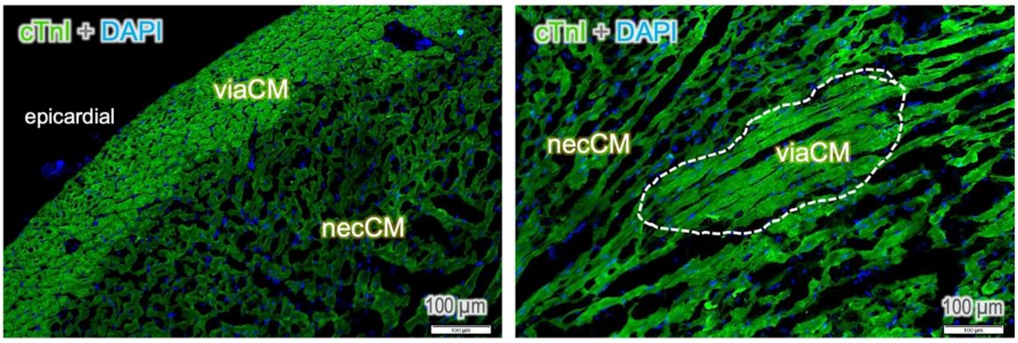

Viability detection by fluorescence intensity in cTnI staining at the very early stage. Heart sections were analyzed as early as 6 hours post-MI. Necrotic tissue exhibited a massive loss of structural integrity, and necrotic cardiomyocytes (necCM) showed diminished—but not completely depleted—fluorescence intensity in cTnI staining. In contrast, viable cardiomyocytes (viaCM) were primarily detected in the sub-epicardial area and occasionally appeared in small clusters within the infarct zone. Notably, discrimination between viaCM and necCM remained challenging at this time point.

Supplementary Figure 4

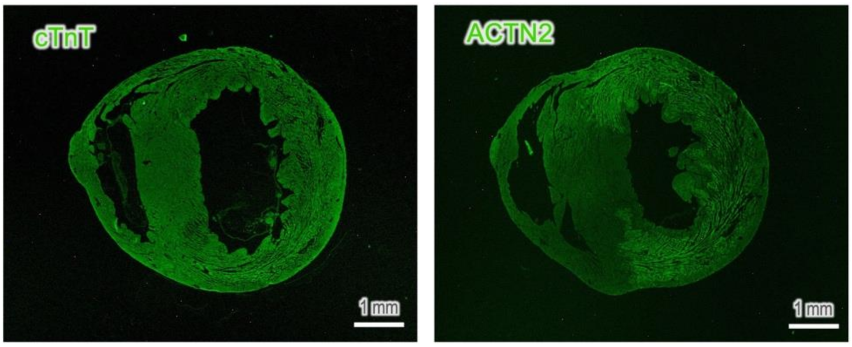

Whole-section images of heart tissue stained by either cTnT or ACTN2. Immunostaining in the heart sections (24 hours post-MI) using anti-cTnT or anti-ACTN2 antibodies reveals strong residual fluorescence signals in the infarct region and a low viable-verse-necrotic signal ratio which makes delineation of the infarct area difficult. Note the hyper-reactivity caused by the necrotic myocardium in the infarcted anterolateral ventricular wall.
